# Case Report: Successful primary percutaneous coronary intervention in octogenarian with acute-on-chronic kidney disease and total atrioventricular block after acute myocardial infarction

**DOI:** 10.12688/f1000research.51858.2

**Published:** 2022-02-09

**Authors:** Andrianto Andrianto, Ni Putu Anggun Laksmi, Rio Herdyanto

**Affiliations:** 1 Department of Cardiology and Vascular Medicine, Universitas Airlangga - Dr. Soetomo Hospital, Surabaya, East Java, 60286, Indonesia; 2 Department of Cardiology and Vascular Medicine, Dr. R. Sosodoro Djatikoesomo Hospital, Bojonegoro, East Java, 62119, Indonesia

**Keywords:** primary PCI, dialysis, acute myocardial infarction, STEMI, acute-on-chronic kidney disease

## Abstract

Myocardial infarction (MI) is frequently complicated by the worsening of renal function. Undergoing primary percutaneous coronary intervention (PCI) becomes crucial to a patient with ST-segment elevation myocardial infarction (STEMI). With appropriate management of MI, acute-on-chronic kidney disease (ACKD) requiring dialysis post-MI remains an important clinical predictor of elevated in-hospital mortality among patients with MI.

In this study, we reported an octogenarian patient suffering from STEMI with ACKD and total atrioventricular block (TAVB). She underwent insertion of a temporary pacemaker and primary PCI. Renal function was improved after dialysis by decreasing the amount of serum creatinine from 8.1 mg/dL at admission to 1.05 mg/dL after primary PCI and dialysis. Primary PCI should still be considered for patients with acute MI, even though these patients have kidney disease, to save the heart muscle and even indirectly improve the kidney function itself.

## Introduction

The incidence of acute-on-chronic kidney disease (ACKD) varies among patients with ST-segment elevation myocardial infarction (STEMI), ranging from 5% to 30%. The structural and functional changes in the kidneys leading to chronic kidney disease (CKD) is most likely to be caused by different pathological mechanisms, including renal hypoperfusion, ischemia, and nephrotoxicity. Despite the proper treatment of myocardial infarction, ACKD still leads to a higher risk of morbidity and mortality, particularly when dialysis is required. ^1^

Octogenarian patients were rarely included in clinical investigations. Patients of advanced age possess the dilemma of revascularization therapy. Many clinicians fear that the risk-benefit ratio of doing the primary PCI is not worth it on elderly patients. ^2^ Fox et al. states the risk of death in patient with ACS is increasing with age, but elderly patients may get an equal or greater benefit from primary PCI. ^3^

## Case presentation

An 80-year-old Asian woman, with no history of smoking and unremarkable medical history, was referred to the emergency room department with a complaint of typical chest pain. The pain began four hours before hospitalization. It further radiated to the back and was accompanied by cold sweats. In this regard, the patient had a history of hypertension. Blood pressure was 116/53 mmHg, pulse rate was 39 ×/min, respiration rate was 23 ×/min, body temperature was 36.7°C, and oxygen saturation was 96% with O _2_ nasal cannula 3 l/min. Her extremities were warm, with vesicular pulmonary arteries and with no crackles or wheezing.

**Figure 1.**
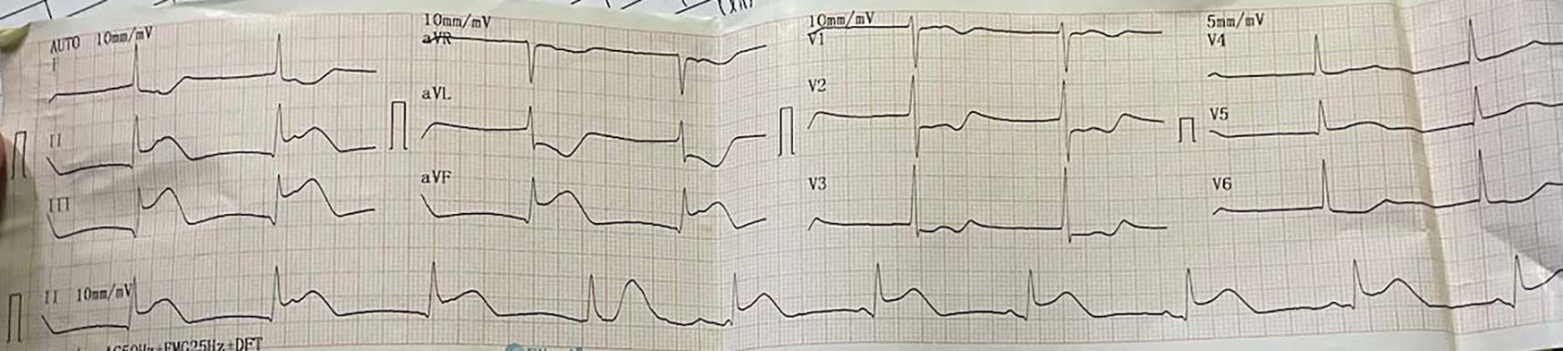
Initial presentation of electrocardiogram with inferior ST-elevation myocardial infarction.

The electrocardiogram (ECG) of the patient showed TAVB with a junctional escape rhythm of 46 times per minute and inferior STEMI ( Figure 1). The laboratory results of the initial visit were within normal limits, except that the blood urea nitrogen (BUN) 138 mg/dL (normal range: 8 – 23 mg/dL), creatinine serum 8.1 mg/dL (normal range: 0.6 – 1.3 mg/dL), haemoglobin (Hb) 8.6 g/dl (normal range: 11.5 – 16.5 g/dl) and troponin I increased to 30.6 ng/mL (normal range: ≤ 0.04 ng/mL).

From these data, we provided this patient with a diagnosis of inferior STEMI with total AV block and ACKD. She was administered with aspilet 300 mg and clopidogrel 75 mg per oral. Subsequently, a temporary pacemaker (TPM) was installed using local anesthesia with the heart rate at 70 ×/min, the sensitivity at 3 mV and the output at 3 V, and diagnostic coronary angiography (DCA) was performed.

**Figure 2.**
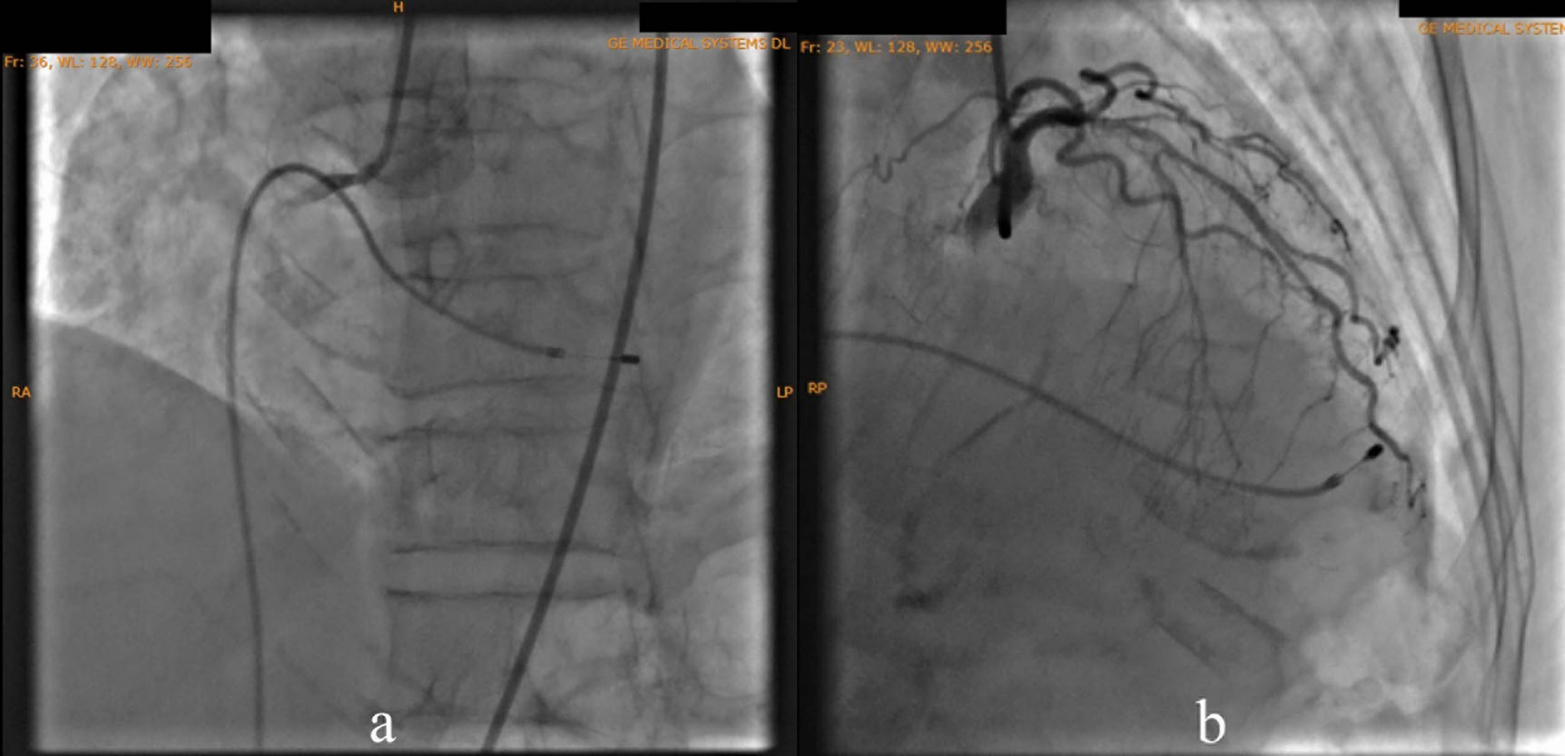
Total occlusion in proximal right coronary artery and 30% in proximal-mid left anterior descending artery.

**Figure 3.**
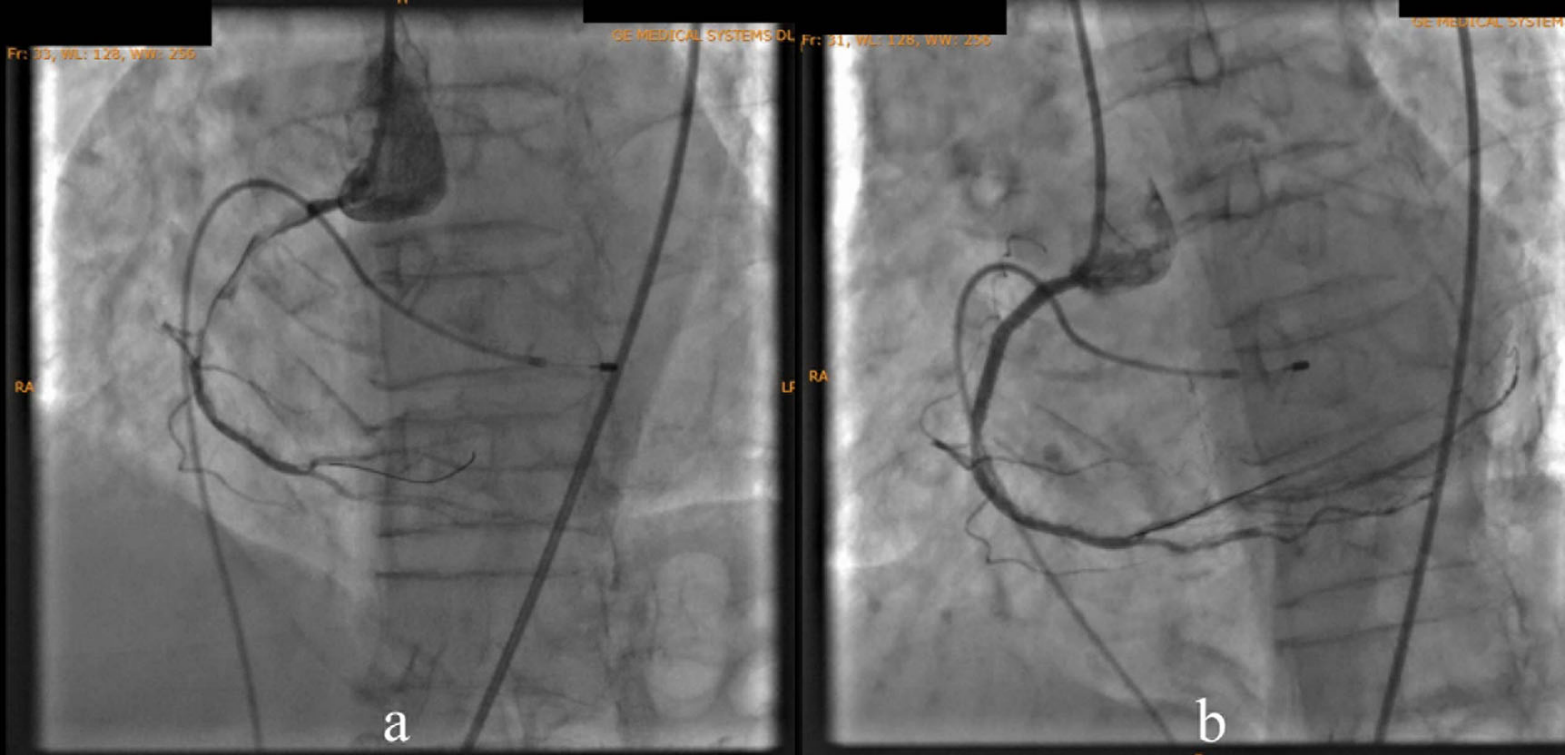
Primary percutaneous coronary intervention at proximal-mid right coronary artery with thrombolysis in myocardial infarction flow 3.

**Figure 4.**
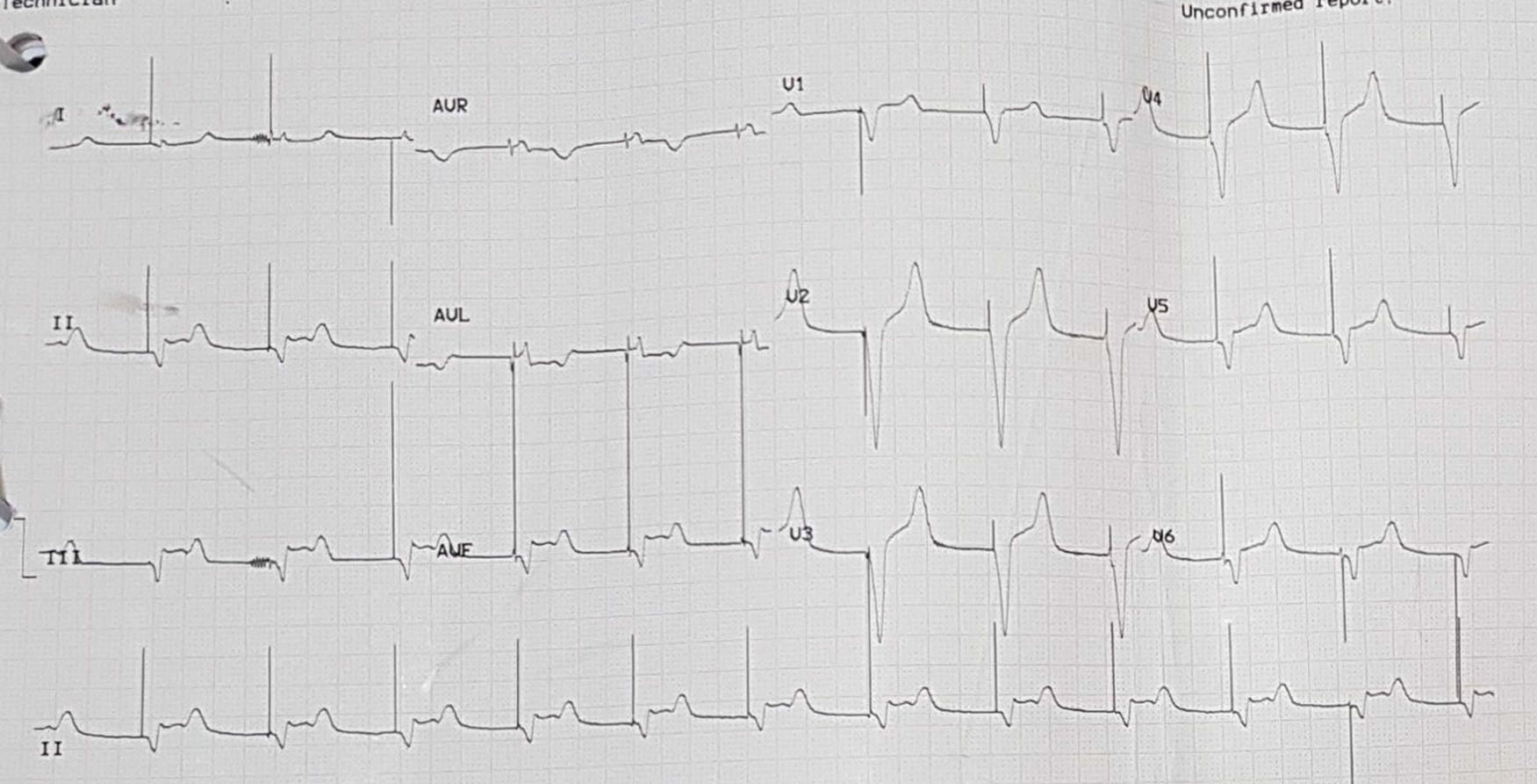
Electrocardiogram after pacemaker installation with rhythm.

According to the DCA, the total occlusion was at the proximal right coronary artery (RCA) ( Figure 2). There was insignificant stenosis in the proximal-mid left anterior descending artery. Besides, there were no stenosis on the left circumflex artery and the left main coronary artery. Primary PCI at the RCA was conducted using drug eluting stent (DES) promus and then thrombolysis in myocardial infarction (TIMI) grade 3 flow was shown at the RCA ( Figure 3). Electrocardiography was also conducted after the installation of the pacemaker ( Figure 4).

**Figure 5.**
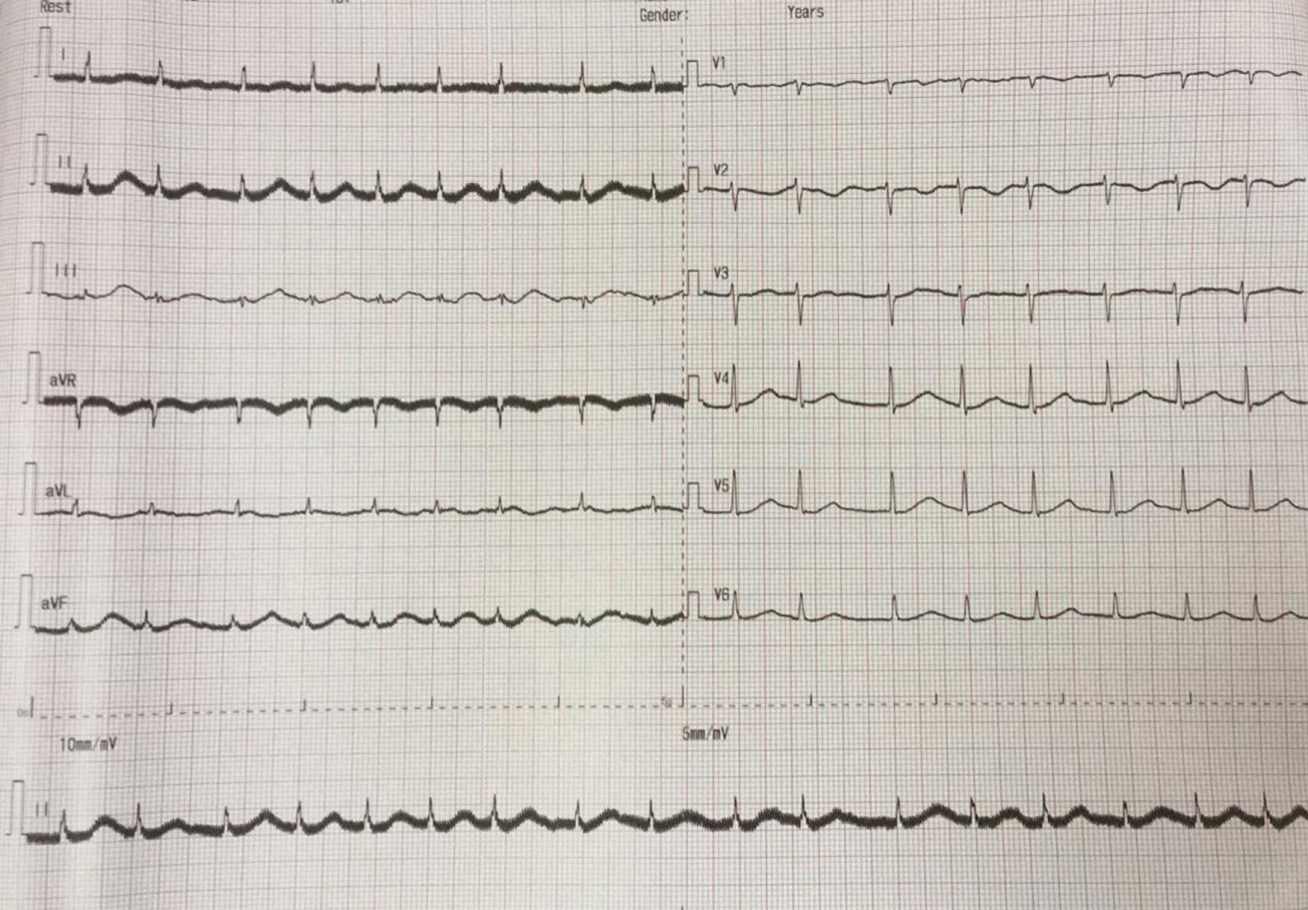
Electrocardiogram of patient in atrial fibrillation condition.

**Figure 6.**
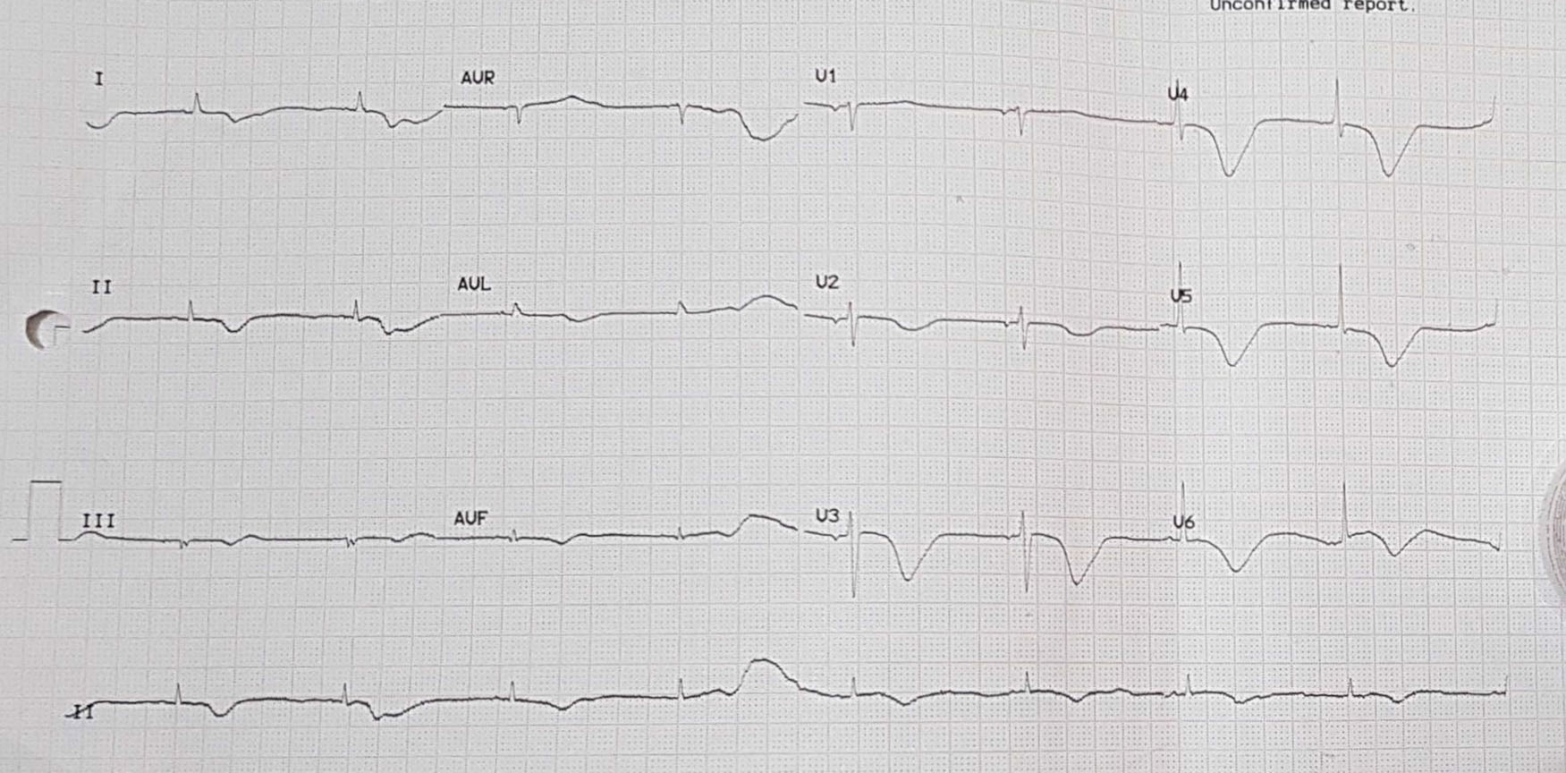
Electrocardiogram of patient with temporary pacemaker off and sinus bradycardia.

The patient was given therapy with dual antiplatelet aspirin 1 × 100 mg per oral and clopidogrel 1 × 75 mg per oral, atorvastatin 1 × 20 mg per oral, N-Acetylcysteine 3 × 200 mg per oral, packed red cell (PRC) transfusion of one bag per day, and TPM. On the third day of the treatment, electrocardiographic imaging indicated that the atrial fibrillation rhythm had a rapid ventricular response of 95–120 times per minute ( Figure 5). As a consequence, the patient was given additional treatment with digoxin 0.25 mg through intravenous infusion. Additionally, she was considered by the internist for haemodialysis (HD). This consideration was made based on the amount of BUN 138 mg/dL (normal range: 8 – 23 mg/dL) and serum creatinine 8.1 mg/dL (normal range: 0.6 – 1.3 mg/dL) from the earlier laboratory results. The internist ultimately decided to administer HD using ultrafiltration 2000 ml to this patient on the fourth day of treatment. The patient did not complain about chest pain on the next (fifth) day. The post-HD laboratory results showed that BUN reduced to 34 mg/dL, serum creatinine to 1.05 mg/dL (eGFR 50 mL/min/1.73 m ^2^). Afterwards, removal of the TPM was attempted on the ninth day ( Figure 6). In this case, the patient was given additional therapy of salbutamol 4 mg per oral three times a day. Later, the patient was discharged from the hospital on the fifteenth day.

**Figure 7.**
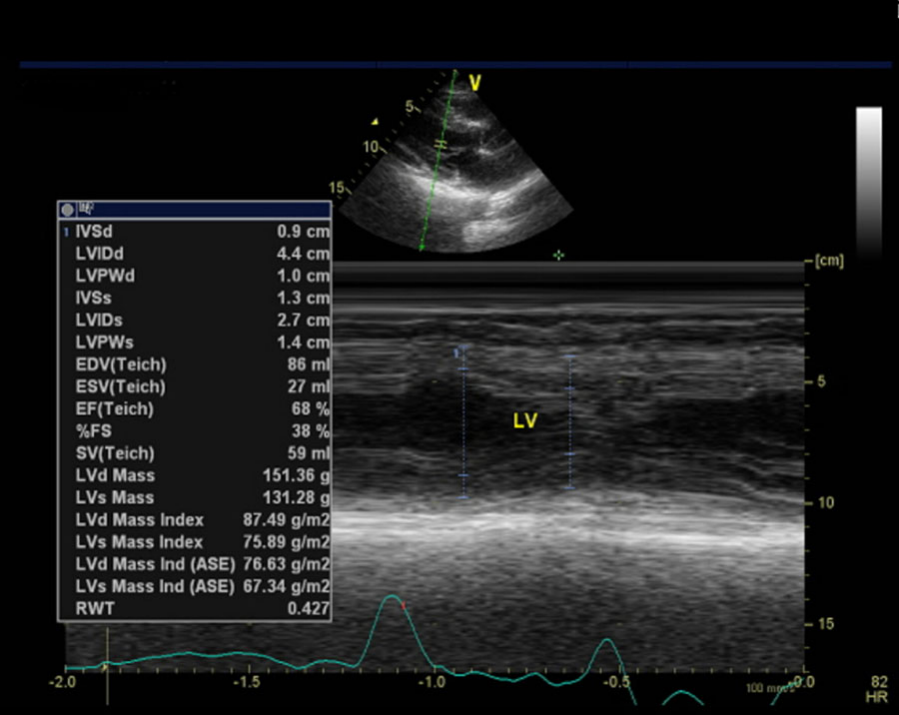
Echocardiography of third week after hospitalization.

After three weeks of leaving the hospital, the patient was asked to undergo echocardiography. The results of this examination demonstrated that there was mild mitral regurgitation with normal cardiac chamber dimensions. The left ventricular systolic function seemed normal with ejection fraction by Teich being 68%, as did the left ventricular diastolic function with E/A being 0.87 ( Figure 7). On the other hand, the right ventricular systolic function appeared normal with tricuspid annular plane systolic excursion accounting for 1.8 cm.

## Discussion

STEMI patients who have previously had kidney diseases or have recently suffered from kidney diseases as a result from STEMI have a worse prognosis. The renal insufficiency of patients with STEMI will increase the mortality and morbidity of cardiovascular diseases. ^4^

Atherosclerotic coronary plaque can cause coronary arterial disease (CAD) in the general population, but for patients with CKD, the pathophysiology of vascular diseases is different, due to a number of new risk factors, e.g., endothelium dysfunction, CKD-related mineral bone diseases, increased oxidative stress, and inflammation. In CKD, atherosclerosis often occurs in form of calcification of the lining of the blood vessels, which is regularly observed on peripheral vessels like the tibial and femoral arteries and on small epicardial blood vessels that contribute to microcirculation. There are many contributing factors to the incidence of chronic inflammation, such as increased oxidation and disrupted antioxidant system. These are particularly linked to hypoalbuminemia and malnutrition. Because of the deterioration of the renal function, the levels of pro-inflammatory cytokines and inflammatory mediators in the plasma will increase, thus leading to blood vessel calcification. ^4^^–^^6^

The recent guidelines recommend that aspirin should be administered immediately after suspected acute coronary syndrome (ACS) and continued without a predetermined time, except if there is contraindication. ^7^ Following that patients with renal insufficiency have an increased risk of bleeding, some doubt exists about the use of therapeutic antiplatelet therapy to such patients. According to the data collected, aspirin is safe and effective for ACS patients with CKD and recommended to be used in these patients to reduce mortality risk and vascular incidence. ^8^

P2Y12 inhibitors are one of the common therapies given to patients with ACS. Various studies have shown the effectiveness of prasugrel and ticagrelor in the management of ACS. Although the risk of bleeding is relatively high, these drugs have a higher potential ratio of decreasing ischemic risk in CKD patients. The lack of alternative therapies related to the interaction of renal function allows clopidogrel to be considered as a treatment in patients with decreased renal function. For patients aged over 75 years old, clopidogrel can be administered at a loading dose of 75 mg and followed with a dose of 75 mg/day for maintenance. ^9^

A number of studies suggested that statins should be used to treat ACS in order to reduce death risk or vascular incidence. American Heart Association guidelines recommend that statins should be given without seeing the initial levels of low-density lipoprotein in ACS patients without any contraindications. ^8^

The use of lipid-lowering therapy, especially with statins, in patients with CKD remains a controversy to date. The potential of a statin-based treatment to decrease the number of vascular events will become much smaller due to a decrease in eGFR. The KDIGO guidelines propose that statins should be used in CKD patients over 50 years old, but not in dialysis patients. This recommendation is mainly based on two studies, namely 4D (Deutsche Diabetes Dialyse Studie) and AURORA (Rosuvastatin use evaluation). Nonetheless, statins still function as the foundation of the lipid management of CKD patients with CAD. In other words, giving statins to ACS patients with CKD is highly recommended. ^4^^,^^8^^,^^10^

Whether patients with the symptoms of CKD and/or end-stage renal disease should be treated with medical therapy or revascularization through either PCI or coronary artery bypass grafting is still debatable. In many cases, STEMI patients with CKD undergo the same invasive treatment as STEMI patients with no CKD. This is adherent to the fact that no specific clinical trial has been conducted of patients with CKD. Although the research appears to be more supportive of initial invasive treatment than of initial conservative treatment, there is no survival benefit from early intervention in CKD patients within a range of Grade 3a to Grade 5 (<60 ml/min/1.73 m ^2^) in non-ST-segment-elevation randomized controlled trials of myocardial infarction. ^10^

There is no contraindication observed in the thrombolytic therapy in this case. Nevertheless, when the worsened CKD and the in-hospital mortality rate are taken into account as a result from myocardial infarction, primary PCI should be chosen. ^10^^–^^12^

Total AV block is common in inferior STEMI and is often transient only and sometimes it can return within 5 to 7 days. Treatment with a temporary pacemaker and PCI is needed to deal with this problem. Meanwhile, the management of STEMI in elderly patients is generally the same as in adult patients. In a retrospective study of 57,000 patients with the acute coronary syndrome (> 75 years old) who received recommended therapy, the in-hospital mortality rate was lower than that of those who did not. ^13^

This case presents a new successfully reperfusion therapy in octogenarian patient with kidney disease. The limitation of this case is that we did not perform further diagnostic tests to find out the underlying disease that had caused the kidney disease.

## Conclusions

The prognosis of patients with decreased renal function and acute myocardial infarction is relatively poor, considering that these two conditions worsen each other. An increased rate of major adverse cardiovascular events, heart failure, and chest pain are seen in line with decreased eGFR rate. In this study, we reported the treatment of a patient of 80 years old suffering from acute myocardial infarction with ACKD and TAVB. The patient, with inferior STEMI, complicated by total AV block, was treated with coronary angioplasty and hemodialysis and those were performed successfully. The complaint of ischemic chest pain was resolved, and renal function was improved. In this regard, serum creatinine decreased from 8 mg/dL to 1.05 mg/dL, with eGFR being 50 mL/min/1.73 m ^2^.

## Data availability

All data underlying the results are available as part of the article and no additional source data are required.

## Consent

Written informed consent for publication of their clinical details and/or clinical images was obtained from the patient.
