# Supplementary material for: Case Report: Successful primary percutaneous coronary intervention in octogenarian with acute-on-chronic kidney disease and total atrioventricular block after acute myocardial infarction
Source: F1000Res. 2022 May 12;10:267. Originally published 2021 Apr 1. [Version 3] doi: 10.12688/f1000research.51858.3 (PMC9123330; doi:10.12688/f1000research.51858.3)
Supplement: Peer review history

# Peer review: Reviewer response for version 3

**DOI:** 10.5256/f1000research.133936.r137601

**Published:** 2022-05-23

**Authors:** Mohammad Ullah Firoze

**Affiliations:** 1 Department of Cardiology, Dhaka Medical College, Dhaka, Bangladesh

The article can be accepted for indexing.

Are enough details provided of any physical examination and diagnostic tests, treatment given and outcomes?

Partly

Is the case presented with sufficient detail to be useful for other practitioners?

Partly

Is sufficient discussion included of the importance of the findings and their relevance to future understanding of disease processes, diagnosis or treatment?

No

Is the background of the case’s history and progression described in sufficient detail?

Partly

Reviewer Expertise:

My area of interest is cardiovascular medicine.

I confirm that I have read this submission and believe that I have an appropriate level of expertise to confirm that it is of an acceptable scientific standard.

# Peer review: Reviewer response for version 3

**DOI:** 10.5256/f1000research.133936.r137602

**Published:** 2022-05-20

**Authors:** Dike B. Ojji

**Affiliations:** 1 Department of Medicine, Faculty of Clinical Sciences, University of Abuja, Abuja, Nigeria

The authors have addressed my initial concerns and I am recommending that the manuscript be accepted for indexing.

Are enough details provided of any physical examination and diagnostic tests, treatment given and outcomes?

Yes

Is the case presented with sufficient detail to be useful for other practitioners?

Yes

Is sufficient discussion included of the importance of the findings and their relevance to future understanding of disease processes, diagnosis or treatment?

Yes

Is the background of the case’s history and progression described in sufficient detail?

Yes

Reviewer Expertise:

NA

I confirm that I have read this submission and believe that I have an appropriate level of expertise to confirm that it is of an acceptable scientific standard.

# Peer review: Reviewer response for version 2

**DOI:** 10.5256/f1000research.120591.r123006

**Published:** 2022-02-16

**Authors:** Mohammad Ullah Firoze

**Affiliations:** 1 Department of Cardiology, Dhaka Medical College, Dhaka, Bangladesh

Thank you. Authors have tried to answer most of the questions and had made some corrections. I think this can be indexed as a case report.

Are enough details provided of any physical examination and diagnostic tests, treatment given and outcomes?

Partly

Is the case presented with sufficient detail to be useful for other practitioners?

Partly

Is sufficient discussion included of the importance of the findings and their relevance to future understanding of disease processes, diagnosis or treatment?

No

Is the background of the case’s history and progression described in sufficient detail?

Partly

Reviewer Expertise:

My area of interest is cardiovascular medicine.

I confirm that I have read this submission and believe that I have an appropriate level of expertise to confirm that it is of an acceptable scientific standard.

# Peer review: Reviewer response for version 1

**DOI:** 10.5256/f1000research.55068.r89511

**Published:** 2021-11-11

**Authors:** Dike B. Ojji

**Affiliations:** 1 Department of Medicine, Faculty of Clinical Sciences, University of Abuja, Abuja, Nigeria

In this case report the authors report a case of  an octogenarian patient suffering from STEMI with ACKD and total atrioventricular block (TAVB), who underwent insertion of a temporary pacemaker and primary PCI.

 Patient also had improved renal function after dialysis by decreasing the amount of serum creatinine from 8.1 mg/dL at admission to 1.05 mg/dL after primary PCI and dialysis. 

 This is an interesting, but the authors have to address some issues to make it more interesting:

- Majorly, the discussion is too broad and was not directly in reference to the case study. Since this is a case report the the findings in this patients should be cited  throughout the discussion section.
- On the minor side:
  - I think the statement in the case report session: 'In this regard, the patient had a history of hypertension' is  is not clear and need to be rephrased.
  - History hypertension was mentioned but BP  was 116/53mmHg. Is patient on any anti-hypertensives.
  - What is meant by the statement: 'we provided this patient with a diagnosis of inferior STEMI with total AV block and ACKD. What is meant by provided'. I think this has to be rephrased.

Are enough details provided of any physical examination and diagnostic tests, treatment given and outcomes?

Yes

Is the case presented with sufficient detail to be useful for other practitioners?

Yes

Is sufficient discussion included of the importance of the findings and their relevance to future understanding of disease processes, diagnosis or treatment?

Yes

Is the background of the case’s history and progression described in sufficient detail?

Yes

Reviewer Expertise:

Preventive Cardiology; Hypertension; Hypertensive Heart Failure; Echocardiography

I confirm that I have read this submission and believe that I have an appropriate level of expertise to state that I do not consider it to be of an acceptable scientific standard, for reasons outlined above.

# Peer review: Reviewer response for version 1

**DOI:** 10.5256/f1000research.55068.r89510

**Published:** 2021-07-26

**Authors:** Mohammad Ullah Firoze

**Affiliations:** 1 Department of Cardiology, Dhaka Medical College, Dhaka, Bangladesh

This is a nice case report to focus on the issue of primary PCI in octogenarians and in patients with renal failure. It’s not a new method or rare event, but it will draw attention of the readers to this issue. But it needs some correction and clarifications:

1. Figure 1 is showing sinus bradycardia not CHB. It should be corrected.
2. Total occlusion is better seen in first figure of Fig-3. So, it should be the first figure of Fig 2. And the first figure of Fig 2 should be the first figure of Fig 3. These two figures should be swapped.
3. N-Acetylcysteine was given in the form fluimucyl. It should be mentioned as generic name.
4. Why was daily packed red cell transfusion given?
5. Was the addition of anticoagulation considered during AF?
6. Why was digoxin used in ACS with AF with renal failure instead of beta blocker or amiodarone?
7. The ECG after removal of TPM there was sinus bradycardia with a rate of 52/min and QTc of 559 msec and t wave inversions. What was the rationale for using salbutamol? What was the possible cause of prolong QTc with T wave inversion? Was there any electrolyte imbalance? What was the rhythm and rate during discharge?
8. What was the baseline echo during admission? What was the possible cause of moderate MR at three weeks? What was the renal function and ECG status at three weeks after hospital discharge?
9. Discussion- PPCI in octogenarians and management of CHB in ACS could be discussed.
10. CIN could be discussed.
11. Justification of hemodialysis should also be discussed. Whether it was done only because of high creatinine level? And what was the creatinine level on the fourth day before starting haemodialysis? Haemodialysis for contrast clearing is not recommended. What was the creatinine level on the day of discharge?
12. This is not a new reperfusion therapy- as mentioned in discussion.
13. Conclusion- It could be more concise. 4 ^th^ & 5 ^th^ sentence could be single sentence. 6 ^th^ & 7 ^th^ sentence are not necessary. Rather a recommendation from the authors could be added.
14. Either CHB or TAVB – anyone of them should be used in the text.

Are enough details provided of any physical examination and diagnostic tests, treatment given and outcomes?

Partly

Is the case presented with sufficient detail to be useful for other practitioners?

Partly

Is sufficient discussion included of the importance of the findings and their relevance to future understanding of disease processes, diagnosis or treatment?

No

Is the background of the case’s history and progression described in sufficient detail?

Partly

Reviewer Expertise:

My area of interest is cardiovascular medicine.

I confirm that I have read this submission and believe that I have an appropriate level of expertise to confirm that it is of an acceptable scientific standard, however I have significant reservations, as outlined above.

# Response

**Published:** 2022-01-23

**Authors:** Andrianto Andrianto

**Affiliations:** Department of Cardiology and Vascular Medicine, Faculty of Medicine, University of Airlangga, Soetomo Academic and General Hospital, Indonesia

1. Figure 1 is showing sinus bradycardia not CHB. It should be corrected.

***Answer:*** Thank you very much for the correction. We have attached the ECG result during inferior STEMI with TAVB in this patient.

 2. Total occlusion is better seen in first figure of Fig-3. So, it should be the first figure of Fig 2. And the first figure of Fig 2 should be the first figure of Fig 3. These two figures should be swapped.

***Answer:*** Thank you very much. We will revise it.

 3. N-Acetylcysteine was given in the form fluimucyl. It should be mentioned as generic name.

***Answer:*** Thank you very much. We have amended it.

 4. Why was daily packed red cell transfusion given? 

***Answer:*** Packed red cell transfusion was administered due to anemia (Hb 8.6 g/dL).

 5. Was the addition of anticoagulation considered during AF? 

***Answer:*** The patient had paroxysmal atrial fibrillation, and, at that time, we did not consider adding an anticoagulant, but we considered the high risk of bleeding in the elderly.

 6. Why was digoxin used in ACS with AF with renal failure instead of beta blocker or amiodarone? 

***Answer: *** Because there were no intravenous beta-blockers in the hospital at that time. Thus, it was digoxin that could be considered. Patients with stage 5 chronic kidney disease (glomerular filtration rate [GFR] < 15 mL/minute), or for those on hemodialysis, alternative agents (e.g., beta-blockers or nondihydropyridine calcium channel blockers) may be preferred for heart rate control; however, it is recommended to reduce the loading dose of 3 to 5 mcg/kg (0.25 to 0.375 mg) should digoxin be used.

 7. The ECG after removal of TPM there was sinus bradycardia with a rate of 52/min and QTc of 559 msec and t wave inversions. What was the rationale for using salbutamol? What was the possible cause of prolong QTc with T wave inversion? Was there any electrolyte imbalance? What was the rhythm and rate during discharge?

***Answer: *** Well, thank you very much. As a beta-agonist, Salbutamol is expected to have a positive chronotropic effect in this patient. Yes, the patient had an electrolyte imbalance, namely hypokalemia of 2.8 meq/l, which had been corrected. On discharge, the patient had asymptomatic sinus bradycardia at 55 bpm.

 8. What was the baseline echo during admission? What was the possible cause of moderate MR at three weeks? What was the renal function and ECG status at three weeks after hospital discharge?

***Answer: *** We apologize for the mistake. There was mild mitral regurgitation on the patient's echocardiography, instead of moderate MR. The renal function was improved after hospital discharge. BUN was 34 mg/dL (normal range: 8–23 mg/dL), and serum creatinine was 1.05 mg/dL (normal range: 0.6–1.3 mg/dL).

 9. Discussion- PPCI in octogenarians and management of CHB in ACS could be discussed.

***Answer: *** Thank you very much. We will include your suggestions in the revised article.

 10. CIN could be discussed.

***Answer: *** Thank you very much for your advice.

 11. Justification of hemodialysis should also be discussed. Whether it was done only because of high creatinine level? And what was the creatinine level on the fourth day before starting haemodialysis? Haemodialysis for contrast clearing is not recommended. What was the creatinine level on the day of discharge?

***Answer: *** Yes, it was done because of the high level of creatinine (8.1 mg/dL) and BUN (138 mg/dL). The creatinine level on the fourth day before starting hemodialysis was 5.8 mg/dl and BUN 109 mg/dl. On discharge, BUN was 34 mg/dL, and serum creatinine was 1.05 mg/dL. 

 12. This is not a new reperfusion therapy- as mentioned in discussion.

***Answer: *** We are very thankyou for the advise.

 13. Conclusion- It could be more concise. 4th & 5th sentence could be single sentence. 6th & 7th sentence are not necessary. Rather a recommendation from the authors could be added.

***Answer: *** Thank you very much for your advice.

 14. Either CHB or TAVB – anyone of them should be used in the text.

***Answer: *** Thank you. We use the term Total AV block.
